# Study of the Potential Hepatoprotective Effect of Myo-Inositol and Its Influence on Zebrafish Development

**DOI:** 10.3390/nu13103346

**Published:** 2021-09-24

**Authors:** Tomasz Antonowski, Karol Wiśniewski, Piotr Podlasz, Adam Osowski, Joanna Wojtkiewicz

**Affiliations:** 1 Department of Human Physiology and Pathophysiology, School of Medicine, Collegium Medicum, University of Warmia and Mazury, 10-082 Olsztyn, Poland; adam.osowski@uwm.edu.pl (A.O.); joanna.wojtkiewicz@uwm.edu.pl (J.W.); 2 Students’ Scientific Club of Pathophysiologists, Department of Human Physiology and Pathophysiology, School of Medicine, University of Warmia and Mazury, 10-082 Olsztyn, Poland; wisniewski.karol@op.pl; 3 Department of Pathophysiology, Forensic Veterinary Medicine and Administration, Faculty of Veterinary Medicine, University of Warmia and Mazury, 10-719 Olsztyn, Poland; piotr.podlasz@uwm.edu.pl

**Keywords:** myo-inositol, zebrafish, hepatoprotection, ethanol, alcoholic liver disease, acute liver damage

## Abstract

Inositol is a natural substance found widely in plants. It is used in therapies for many medical cases. The aim of this study was to determine the toxicity of myo-inositol (MI) and to investigate its potential hepatoprotective character. In the first part of the study, zebrafish embryos were incubated with 5, 10, 20, 40, 60, 80, and 100 mg/mL MI. Endpoints such as survivability, hatching rate, malformation, and mobility were evaluated. Our results demonstrated that the high doses of MI lead to increased mortality and malformations and reduce the hatching rate in comparison to the control group. Moreover, low doses of this compound do not produce a negative effect on zebrafish and even have the ability to increase the hatching rate and mobility. In the second part of the study, the hepatoprotective effect of MI was tested. Zebrafish larvae from the line Tg (fabp10a:DsRed) were incubated for 24 h with 1% and 2% ethanol (EtOH), 5 mg/mL of MI with 1% EtOH, and 5 mg/mL of MI with 2% EtOH. No significant differences between the groups with EtOH and the group treated with EtOH with MI were observed. Our results suggest that MI has no positive benefits on hepatocytes of zebrafish larvae.

## 1. Introduction

The human liver is an essential organ that performs a large number of physiological functions. It is the primary site of synthesis for a number of serum proteins, such as albumin, prealbumin, plasma fibrinogen, transferrin, transport proteins, phospholipids, and essential fatty acids. The liver plays a role in nutrient metabolism and is the major detoxification organ. In humans, the liver is targeted by developmental, infectious, immune-mediated, metabolic, and neoplastic diseases, all of which are associated with morbidity. In comparison to other organs, the liver has a remarkable regenerative potential [1]. However, this capacity is finite and often inadequate in compensating for severe acute or chronic injuries [2]. An association between many liver diseases and alcohol abuse was recognized more than 200 years ago. Excessive alcohol consumption is a global healthcare problem with enormous economic, clinical, and social consequences. The liver shows the earliest and the greatest degree of tissue injury from excessive drinking because it is the primary site of ethanol metabolism. This contributes to the development of various types of alcohol-induced liver damage [3,4]. As alcohol is broken down in the liver, a number of potentially dangerous products are generated, such as acetaldehyde or free radicals. These products contribute to alcohol-induced liver damage (more than alcohol itself does). Consequently, the symptoms of liver damage may not appear until the organ’s damage is extensive [2,4]. When dealing with chronic liver injuries, due to the obesity pandemic, nonalcoholic fatty liver disease with its main driver, i.e., insulin resistance, should always be taken into account, even though its inner mechanisms are far from clear as evident in the article from 2020 by Tarantino et al. [5].

**Figure 1:**
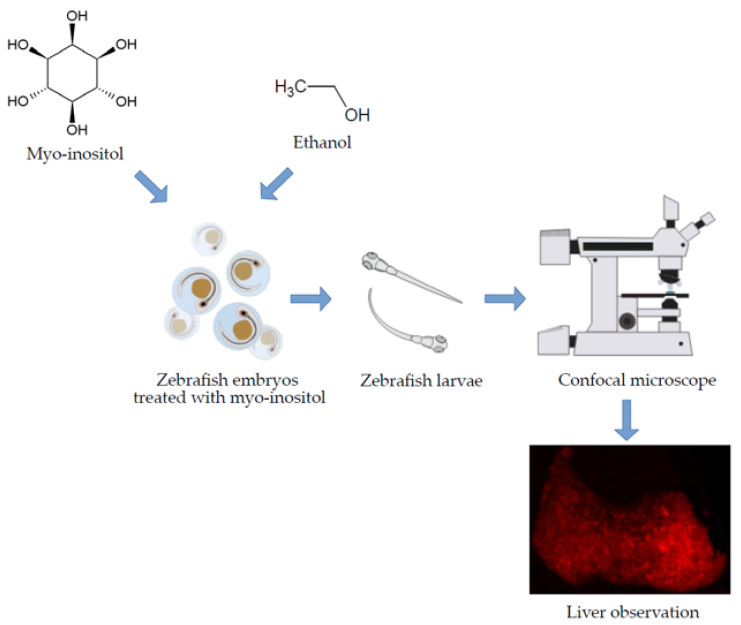
Zebrafish as a model of alcoholic liver disease.

The positive effects of many natural substances from plants on human health have been known for many years. Studies from recent years have shown that increased consumption of vegetables and fruits can reduce the risk of development of many diseases [6]. Cyclitols are gaining more and more interest because they are widespread in the world of plants, can be found in the seeds of cereal grains, legumes (beans), buckwheat, citrus fruits, nuts, wheat germs, and yeast and have a wide range of biological activities. They play a particularly important role in the functioning of the cell; they are involved in the processes of phosphate storage, membrane biogenesis, signal transduction, cell wall formation, osmoregulation, and show an antioxidant activity [7]. In plants, they are one of the compatible soluble substances formed in response to salt or water stress [8,9]. Myo-inositol (MI) is the most popular of all known cyclitol isomers (Figure 1) [10,11,12]. Deficiencies or abnormalities in inositol metabolism interfere with glucose uptake and cause defects in microvascular areas associated with diabetes [13,14]. This compound is present in all eukaryotic cells and acts as a structural basis for a number of transmitters [15]. It is also an important component of structural lipids, such as phosphatidyl inositol [16]. MI induces the conversion of glucose into glycogen and also modulates the activation of glucose transporters and their use [17,18,19]. MI compensates differently for some metabolic deregulations according to insulin resistance; for example, phosphoinositol-3-phosphate, derived from MI, increases glucose transport into cells by stimulating the glucose transporter type 4translocation into the cell membrane [20]. It needs to be underlined that MI toxicity has not yet been directly investigated [21,22,23]. Some scholars recently retrieved 10 studies on animal models assessing MI or pinitol deficiency or supplementation and one human randomized controlled trial (RTC). Overall, inositols’ deficiency was associated with increased fatty liver in animals. Conversely, inositols’ supplementation in animal models of fatty liver reduced hepatic triglycerides and cholesterol accumulation and maintained a normal ultrastructural liver histopathology. In the one included RCT, pinitol supplementation obtained similar results. Pinitol significantly reduced liver fat, postprandial triglycerides, AST levels, and lipid peroxidation increasing glutathione peroxidase activity, as detailed in the article from 2020 by Pani et al. [24].

Zebrafish is a useful model, which provides researchers with the ability to conduct detailed embryological and genetic analyses. In hepatology studies, zebrafish is considered a model representative of human liver and that is why this makes it a particularly useful system for studying the development and morbidity of this organ [25]. Zebrafish are relatively inexpensive to maintain and breed, and hundreds to thousands of embryos can be obtained for analyses in a relatively short time. Their embryos develop externally and are optically transparent during the first weeks of life (Figure 1). This allows direct observations of liver development throughout organogenesis with the use of light and fluorescence microscopy. Zebrafish embryos and larvae develop rapidly, with progression from embryos to free-swimming preying larvae in the fifth day after fertilization [26]. In this developmental stage, the liver comprises well-differentiated hepatocytes coupled to the network of intrahepatic biliary channels. It is worth emphasizing that a zebrafish develops a functional liver on the fourth day after fertilization. Despite some architectural differences, the zebrafish liver contains a parenchymal and stromal cell inventory similar to that of humans. Moreover, hepatocyte synthetic and secretory functions are well developed at this stage. The most commonly used laboratory zebrafish strains are outbred, which helps to avoid the strain-specific effects in comparison to inbred rodent strains [26]. Moreover, it should be mentioned that zebrafish larvae develop signs of alcoholic liver disease (ALD), including steatosis, but sterol response element binding protein transcription factors’ activation is required for steatosis in this model. The tractability of zebrafish genetics provides a valuable tool for dissecting the molecular pathogenesis of acute ALD, as found in Passeri et al. [27].

The anatomical structure, physiology, and metabolic processes of zebrafish liver have revealed multiple similarities with mammals, including humans. A good example is the presence of a gallbladder in zebrafish, which is absent in a laboratory mouse, for example. However, it has to be taken into account that the liver in zebrafish, especially in the larval form, has a high regenerative capacity, while the human liver’s ability to regenerate is limited. Finally, it should be noted that this animal model does not completely mirror human disease, even though it has great value.

## 2. Materials and Methods

### 2.1. MI Toxicity Studies on Zebrafish Larvae

Wild zebrafish strain (Tu–Tuebingen) was set for spawning in spawning containers. Eggs in the stadium 0.25–1 h post-fertilization were collected and selected. Next, two 6-well culture plates were prepared for 8 test groups, for 50 individuals in each well. Then an earlier prepared dose of MI (Chemat, Gdańsk, Poland) solution was added to each well. The following concentrations of the tested compound were prepared: 0 mg/mL (as a control group with E3 zebrafish embryo medium: 5 mM NaCl, 0.17 mM KCl, 0.33 mM CaCl_2_·H_2_O, 0.33 mM MgCl_2_·6H_2_O, and pH 7.2), 5 mg/mL, 10 mg/mL, 20 mg/mL, 40 mg/mL, 60 mg/mL, 80 mg/mL, and 100 mg/mL. The maximum volume of solutions in each well was 5 mL. MI solutions were prepared by dissolving in E3. Plates were incubated at 28.5 °C for 96 h. After every 24 h, solutions in all wells were changed. At 24 hpf, 48 hpf, 72 hpf, and 96 hpf, survival, hatching, and morphology in all groups were tested. Additionally, at 24 hpf, the movement of individuals in all groups was investigated using DanioScope (Noldus Software, Wageningen, The Netherlands) [28].

### 2.2. Hepatoprotective Influence of MI in Acute Liver Damage Zebrafish Larvae 

Zebrafish from line Tg(fabp10a:DsRed) with typical presence of fluorescent protein in the liver were out crossed with wildtype Tu strain. At 96 hpf, larvae showing fluorescence in the liver were selected. Then they were randomly selected for each test group. Larvae were placed in two 6-well plates (15 larvae in each well). Next E3, MI, and ethanol solutions were added immediately. The tested groups were prepared as follows: control group with E3, 5 mg/mL MI solution, 1% ethanol, 2% ethanol, 5 mg/mL of MI with 1% ethanol, and 5 mg/mL of MI with 2% ethanol. The plates were then incubated in 28.5 °C for 24 h. Next, all larvae in each subgroup were fixed in 4% paraformaldehyde overnight in 4 °C. After, fixation samples were washed three times in phosphate-buffered saline with 25%, 50%, and 80% glycerol content.

### 2.3. Imaging and Data Analysis 

Zebrafish larvae from line Tg(fabp10a:DsRed) with DsRed fluorescence protein were analysed under a confocal microscope (ZEISS LSM 710, Jena, Germany). For direct comparison in the same set of experiments, images were taken for the same settings. Calculations were performed on 30 stacked images and cover the image of the entire liver (200 µm) of each photographed larvae. Stacks of images were compiled to produce maximum intensity projection images. ImageJ software was then used to define fluorescence intensity and liver size. All results were analyzed by using the *t*-test in GraphPad Prism 6 (GraphPad Software, Inc., San Diego, CA, USA).

## 3. Results

### 3.1. The Toxicity of Various Concentrations of MI on Zebrafish Model

**Figure 2:**
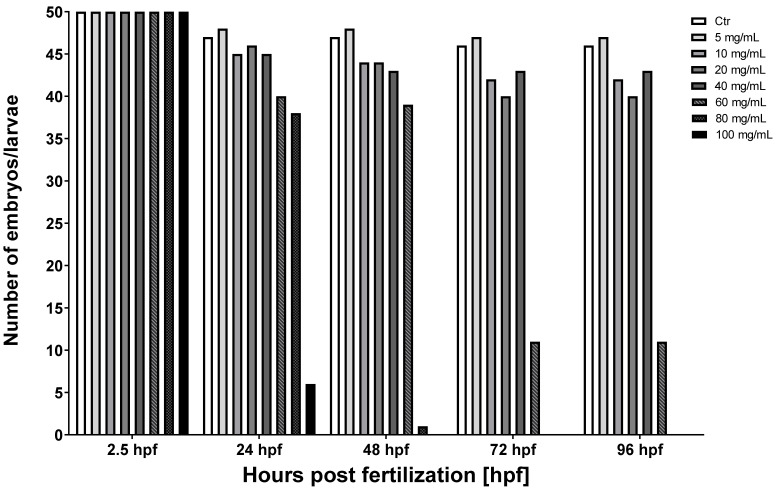
Results of the effect of MI on the zebrafish survival. Ctr–control group; zebrafish embryos were treated by elevating concentrations of MI solutions from 5 to 100 mg/mL. Effect of different concentrations of MI were observed after 24, 48, 72, and 96 h post-fertilization (hpf).

Exposure to MI had significant effects on the survival of zebrafish embryos and larvae. The mortality after 24, 48, 72, and 96 h post-fertilization (hpf) incubations grew significantly with increasing concentration of MI (Table 1) (Figure 2).

**Figure 3:**
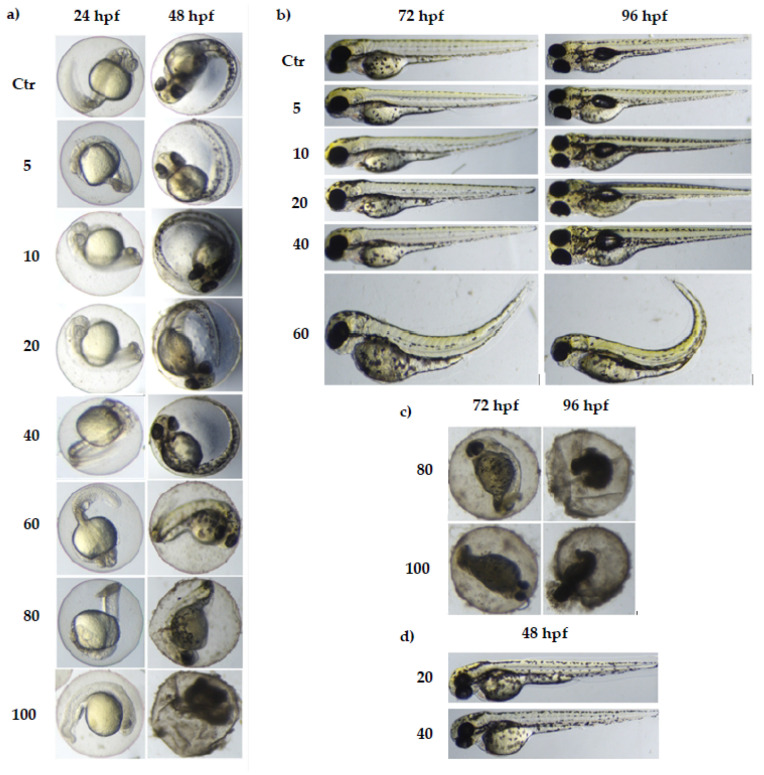
Stages of zebrafish development after incubation in different concentrations of MI. Ctr–control group; zebrafish model was incubated in different concentrations of MI solutions from 5 to 100 mg/mL. Effects were observed after 24, 48, 72, and 96 h post-fertilization (hpf) incubation: (**a**) zebrafish development in control group and in the presence of MI at 24 and 48 hpf (MI concentrations changed from 5 to 100 mg/mL); (**b**) zebrafish development in control group and in the presence of MI at 72 and 96 hpf (MI concentrations changed from 5 to 60 mg/mL); (**c**) zebrafish development in the presence of MI at 72 and 96 hpf (MI concentrations: 80 and 100 mg/mL); (**d**) zebrafish development in the presence of MI at 48 hpf (MI concentrations: 20 and 40 mg/mL).

**Figure 4:**
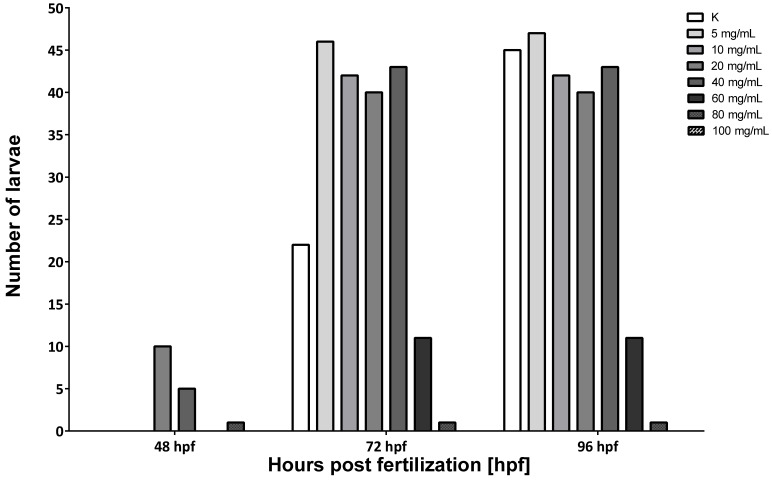
Results of the effect of MI on the zebrafish hatching. Ctr–control group; zebrafish embryos were treated with different concentrations of MI solutions from 5 to 100 mg/mL. Effect of different concentrations of MI were observed at time 48, 72, and 96 h post-fertilization (hpf).

The survival rate began to decline from 24 hpf in 60, 80, and 100 mg/mL doses. The significant rate of survival decreased in the group treated with 80 mg/mL in 72 hpf. Moreover, in the groups with MI concentrations from 60 to 100 mg/mL, after 96 hpf, individuals with developmental deformities began to appear (Figure 3). Exposure to MI also had a significant impact on the hatching of zebrafish larvae (Figure 4). No hatching was observed after 2.5 and 24 h after the fertilization period. The first hatching was observed after 48 hpf in groups with concentrations of 20 and 40 mg/mL (Figure 3). The next ones were observed at 72 hpf in all groups except doses with MI concentrations 80 and 100 mg/mL. All individuals hatched in solution with 60 mg/mL had a characteristic body curvature deformation (Figure 3). Additionally, at 72 hpf, in the groups treated by concentrations from 5 to 40 mg/mL, the number of hatched zebrafish was two times higher than in the control group (Figure 4).

**Figure 5:**
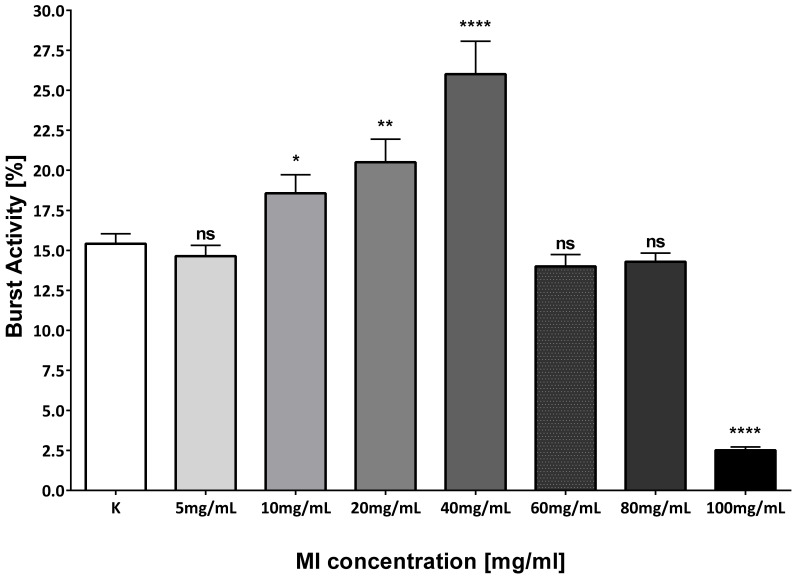
Results of the effect of MI on the zebrafish movement at 24 hpf. Ctr–control group; zebrafish larvae were treated with different concentrations of MI solutions from 5 to 100 mg/mL. Results were analysed by the *t*-test in GraphPad Prism 6. All groups were compared with control group-C. * *p* = 0.0184; ** *p* = 0.0017; **** *p* < 0.0001; ns = nonsignificant.

At 24 hpf after exposure with different MI concentrations, the movement of the individuals also was checked (Figure 5). The highest activity was characteristic for the group treated with 40 mg/mL of MI, and there were significant differences between the control group and groups treated with 10 and 20 mg/mL of MI. The lowest activity of zebrafish larvae was noticed in the group treated with 100 mg/mL. No significant differences were observed between control group and the groups treated with 60 and 80 mg/mL of MI.

The results from this experiment were used to determine a safe dose (5 mg/mL) of MI needed for further studies of the hepatoprotective potential of this compound for zebrafish larvae.

### 3.2. Hepatoprotective Potential of MI in Alcohol-Induced Acute Liver Damage

**Figure 6:**
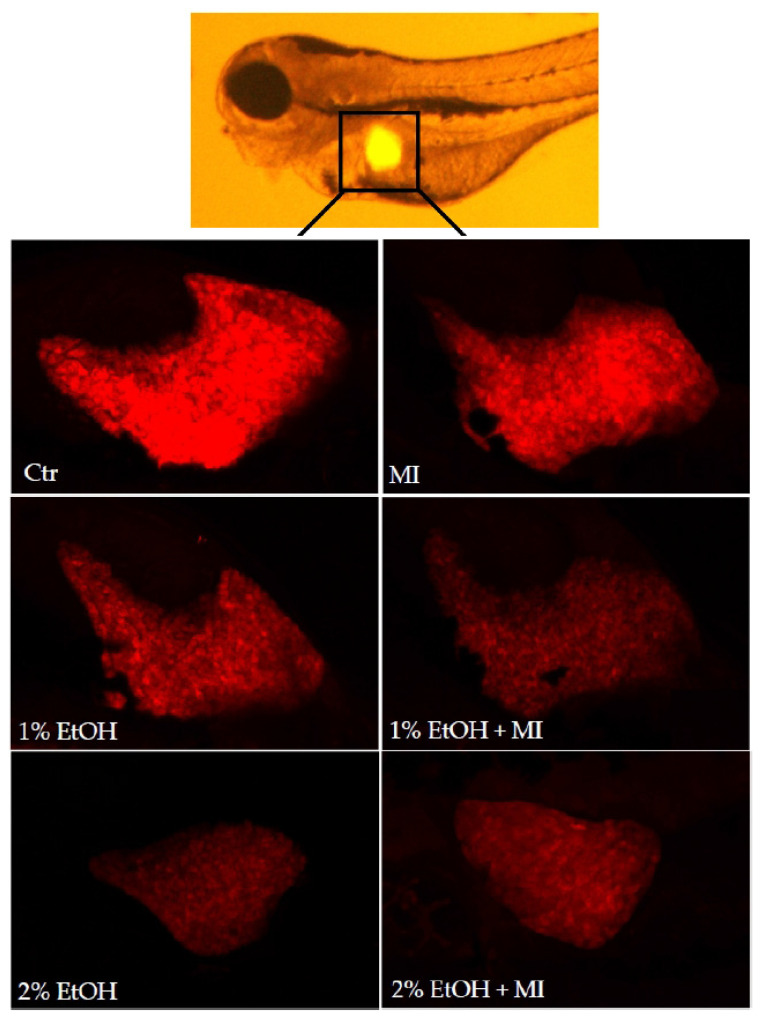
Confocal microscopy images of zebrafish larvae liver.

**Figure 7:**
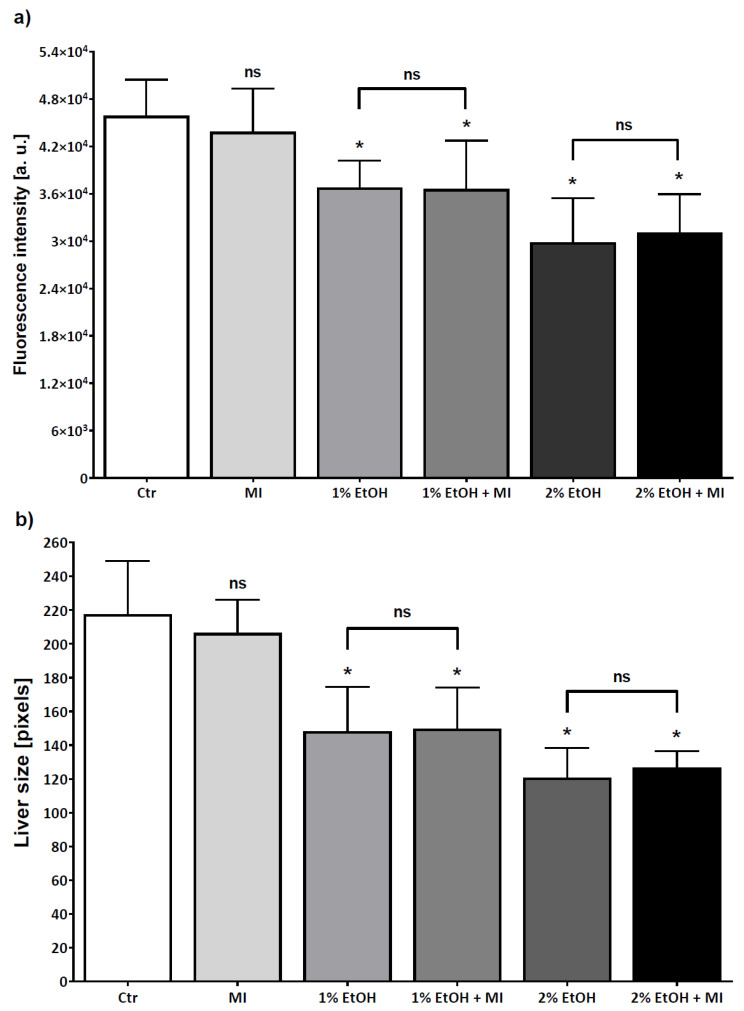
MI protective effect shown by the fluorescence intensity (**a**) and size (**b**) of the liver of zebrafish larvae livers. Results were analysed by the *t*-test in GraphPad Prism 6. All groups were compared with the control group-Ctr. Additionally, groups treated with 1% and 2% of ethanol (EtOH) were compared with groups treated with 1% of ethanol with 5 mg/mL of myo-inositol (MI)and treated with 2% of ethanol with 5 mg/mL of MI, respectively. * *p* < 0.0001; ns = nonsignificant.

MI is a natural compound in medicinal plants that is used for the prevention or treatment of many different diseases such as fatty liver disease, lypo-dystrophy, endothelial dysfunction, metabolic syndrome, polycystic ovary syndrome, and insulin resistance. To evaluate the potential protective effect of MI on hepatocytes, acute liver damage of zebrafish larvae was induced by 1 and 2% ethanol (EtOH). As shown in Figure 6 and Figure 7a, ethanol induced a significant reduction in fluorescence intensity, including groups with ethanol and MI both present in the solution (*p* < 0.0001). Moreover, we examined the liver size, and results suggested that the size in both alcohol concentrations, was significantly lower, even in groups treated by ethanol solution with MI (*p* < 0.0001) (Figure 6 and Figure 7). Additionally, in groups treated with 2% ethanol solution and 2% ethanol with MI, survival of the zebrafish larvae was the lowest in comparison to other groups. As shown in Figure 7, there were no significant differences between the group with 1% EtOH and the group treated by 1% EtOH with 5 mg/mL of MI, and between the group with 2% EtOH and the group treated by 2% EtOH with 5 mg/mL of MI.

## 4. Discussion

Due to their sensitivity to environmental changes, zebrafish larvae are used as a suitable genetic model organism for the monitoring of potentially toxic and teratogenic substances. In the presented study, MI was used to investigate hepatoprotective activity on ethanol induced liver damage. Comparison of the effects of ethanol and MI on embryosled to better understanding the effects of ethanol on early vertebrate development and whether MI could act as a potential protective component against harmful consequences. Natural plant extracts are usually a source of substances that can have a positive effect on different organisms. A diet rich in plant ingredients can contain many physiologically active compounds, such as caffeine, which has a positive effect in alleviating or treating certain diseases [29]. Some plant extracts have become popular drugs, such as Xiaoaiping (XAP), the traditional Chinese medicine from *Marsdenia tenacissima* [30]. This extract consists of aromatic compounds, which are able to induce specific physiological effects [31]. In 2018, Lin J. et al. published an article with the aim of determining the potential toxicity of XAP. In this research, zebrafish embryos were incubated in 0.4, 0.8, 1.2, 1.6, 2.4, and 3.6 mg/mL XAP. Characteristic changes in mortality, hatching rate, and malformation were recorded at 24, 48, 72, 96, and 120 h post-exposition (hpe). The results demonstrated that exposition to XAP reduced the hatching rate and increased the mortality and malformation rate. After addition of XAP, mortality of embryos was recorded in the order with the XAP increasing concentration at 24, 48, 72, 96, and 120 hpe. Similar to our results, a significant increase in mortality was observed in embryo incubation in the highest concentrations of XAP (2.4 and 3.6 mg/mL at 24 and 48 hpe). All fish died after exposure to the highest concentrations of XAP at 72 hpe. At 120 hpe XAP concentrations, 1.6 and 2.4 mg/mL had significantly increased mortality, but the lowest doses, 0.4 and 0.8 mg/mL, had no significant impact on mortality. In our study, exposition to MI had significant effects on the mortality of zebrafish larvae at 24, 48, 72, and 96 hpf. Survivability significantly started to drop from 24 hpf in 60, 80, and 100 mg/mL MI probes, while the significant rate of survival decrease was observed in the group treated with 80 mg/mL of MI in 72 hpf. Additionally, in groups with 80 and 100 mg/mL of MI, none of the zebrafish survived.

Hatching is a very important period in zebrafish embryogenesis and is one of the most important indices of the developmental toxicity evaluation. Embryos exposed to XAP started to hatch after 48 hpe and finished by 96 hpe. The embryo-hatching rate was significantly reduced in groups with 1.6 and 2.4 mg/mL of XAP at 96 hpe and only 25% of embryos hatched by 120 hpe in the group incubated with 2.4 mg/mL of XAP. In our study, exposure to MI, similar to XAP, also had significant effects on zebrafish hatching. The first hatching was observed after 48 hpf in groups with MI concentrations of 40 mg/mL, which was earlier than in the control group (Figure 3 and Figure 4). In other groups, hatching was observed after 72 hpf except probes with 80 and 100 mg/mL MI concentrations. The number of hatching larvae in groups with MI concentrations of 5, 10, and 20 mg/mL was higher than in the control group. In groups with MI concentration 80 mg/mL or higher, all tested animal died, so no hatching was observed.

In both, malformations were observed. Both tested compounds caused characteristic spinal curvature. In the XAP-treated group, this phenomenon was observed in the 1.6 mg/mL at 120 hpe. In our study, spinal curvature began to appear only in the group treated by 60 mg/mL of MI at 72 hpf and 96 hpf (Figure 3). Moreover, the mobility was tested in both studies. XAP-treated zebrafish were characterized by lower mobility. The larvae from the groups with 0.8, 1.2, and 1.6 mg/mL of XAP showed notable decreases in movement. These results demonstrated that XAP impaired the locomotor capacity of zebrafish. In our study, we also checked the mobility at 24 hpf after exposure to different MI concentrations. The highest activity was observed in the group treated by 40 mg/mL of MI only. There were significant differences between groups treated with 10 mg/mL and 20 mg/mL of MI. The lowest activity of zebrafish larvae was noticed in the group treated with 100 mg/mL of MI, and no significant differences were observed between groups treated with 60 and 80 mg/mL of MI (Figure 5) [32]. The obtained differences in hatching and mobility may be due to the fact that XAP in the tested doses may cause damage to the central nervous system. Otherwise, MI in 40 mg/mL concentration, can bind to excitatory receptors in the nervous system. It has been shown that scyllo-inositol, which also belongs to the cyclitol family and is very similar in structure to MI, may have positive neurological properties [33]. Summarizing, MI and XAP have a dose-dependent toxic potential. Both tested compounds have an impact on mortality which depends on their concentration. Additionally, embryos exposed to MI and XAP had a change in the hatching rate.

To investigate whether MI has a hepatoprotective effect in ALD model we used the transgenic line of zebrafish larvae (*Tg(fabp10a:RFP)*) with red fluorescent protein expressed in hepatocytes [34]. Zebrafish larval livers are affected by alcohol in a manner similar to mammals, including changes in liver size, cellular architecture, gene expression patterns, and lipid accumulation [34]. Earlier studies suggest that alcohol is metabolized in zebrafish larval livers, resulting in oxidative stress and steatosis [27] Our results indicated that fluorescence intensity and liver size decrease after exposure to alcohol. Changes in red fluorescence reflect the loss of red fluorescent protein, which may be due to loss of hepatocytes, progressive steatosis, and loss of liver parenchyma [27,34,35].

In 2009, Myung-Sook et al. investigated another member of the cyclitol family, pinitol. It was tested as a potential hepatoprotective agent. Pinitol is a naturally occurring insulin-like substance, which has been demonstrated to be extremely anabolic and has an antihyperglycemic effect as a powerful insulin mimicker. The study included investigations on the hepatoprotective effect of 0.05% (7.7 × 10^−4^ M) and 0.1% (1.05 × 10^−3^ M) levels of pinitol on hamsters with a high cholesterol level induced by a high-fat diet. For comparison, in our study we used a 2.8 × 10^−5^ M concentration of MI. Choi et al. suggested that pinitol supplementation plays an important role in improving lipid. Intake of pinitol significantly lowered the hepatic cholesterol and triglyceride levels. Moreover, pinitol supplementation significantly elevated the hepatic enzymes with antioxidant activities. The authors showed that pinitol significantly reduced the level of H_2_O_2_ and hepatic lipid peroxide production. Accordingly, their data suggest that pinitol is safe and can be an effective material in reducing a hepatic cytotoxicity, which can be induced by a high-fat diet and causes insulin resistance. These results indicate that pinitol has the potential to reduce a hepatotoxic effect caused by a high-fat diet [36]. In our work, no specific hepatoprotective effect of MI was observed. MI and pinitol belong to one biochemical group and MI’s low influence could be the result of the low concentration used in our study, but only this level was tolerated. Moreover, structural differences should be considered, because there is an additional methyl group next to the second carbon in the pinitol structure, which may have a potential impact on the properties of this compound.

In 2019, Xiong et al. investigated the hepatoprotective potential of the *Salvia plebeian* ethanol extracts (SPEE) on zebrafish model as a compound with potential hepatoprotective properties. Similar to plants containing MI, *Salvia plebeia* is a traditional Chinese medicinal herb that has been used for the treatment of many diseases, for example hepatitis. To evaluate the protective effects of SPEE on hepatocytes, acute liver damage was induced in adult zebrafish by 5 mg/mL thioacetamide (TAA) solution. This compound induced a significant increase in hepatic damage (hepatic steatosis) in 72 h. In comparison with the control group, the number of large fat vacuoles in hepatocytes, number of apoptotic cells, necrotic size, stage of liver fibrosis, content of lipid droplets, and inflammatory response were higher in groups treated by TAA. These results showed that in groups of zebrafish treated with TAA and then exposed to 0.5 mg/mL SPEE for 24 h, all hepatic damage was greatly alleviated. Additionally, the authors suggested that ethanol extracts of *Salvia plebeian* can be useful in reducing the negative effects of hepatic steatosis, have the ability to protect hepatic cells against apoptosis caused by chemical compounds, and have an anti-inflammatory effect on adult zebrafish [37]. In our study, no positive effects of protection by MI were observed. The SPEE was used in concentrations ten times lower than those used in our research. Moreover, structural differences should be considered, because SPEE contains four cyclic subunits and MI is a monocyclic compound, but both compounds have the same number of hydroxyl groups. These structural differences may have a potential impact on other properties of these two compounds. It is also worth underlining that in the study of Xiong et al., contrary to ours, adult zebrafish were tested, while we used only zebrafish in the larval stage.

Many studies show that some natural compounds have a potentially positive effect in the treatment of diseases. However, some of these results are contradictory. Therefore, additional studies investigating their potential positive effects are needed. In the present work, we showed that MI at low doses does not possess any protective effect on the zebrafish model of ethanol induced acute liver damage. This compound is widely used in traditional Chinese medicine and has been tested in many scientific studies. We used ethanol damaged liver models to study MI’s influence because EtOH is responsible for many cases of acute liver damage worldwide. Presented results suggest that attempts at MI applications in acute liver damage can be ineffective.

## 5. Conclusions

In summary, many natural substances with potential positive benefits for human health can be found in plants. In the present study, we investigated the influence of MI on zebrafish larvae. MI caused a dose-dependent increase in developmental damage in zebrafish embryos, indicated by an increase in mortality and malformation, such as spinal curvature and a delayed hatching period. We also showed that MI did not show a hepatoprotective effect in the systems we tested. This was probably caused by the low concentrations of MI. Nevertheless, it should be considered that the group of cyclitols is very numerous and conducted research opens the way for further studies. Our study can be helpful in explaining the mechanisms of possible developmental damage caused by cyclitols as well as in determining the hepatoprotective potential of the family of these components.

## Figures and Tables

**Table 1:** Number of zebrafish embryos (2.5, 24, and 48 hpf) and larvae (72 and 96 hpf) survival after MI exposition. Ctr–control group; individuals were treated by elevating concentrations of MI solutions from 5 to 100 mg/mL. Effects of different concentrations of MI were observed after 2.5, 24, 48, 72, and 96 h post-fertilization (hpf).

MI Dose [mg/mL]	2.5 hpf	24 hpf	48 hpf	72 hpf	96 hpf
Ctr	50	47	47	46	46
5	50	48	48	47	47
10	50	45	44	42	42
20	50	46	44	40	40
40	50	45	43	43	43
60	50	40	39	11	11
80	50	38	1	0	0
100	50	6	0	0	0

## Data Availability

Data available on request from the authors.

## Abbreviations

MI	myo-inositol
EtOH	ethanol
hpf	hour post-fertilization
hpe	hour post-exposition
XAP	Xiaoaiping
SPEE	ethanol extracts of *Salvia plebeia* R. Br.
TAA	thioacetamide
RCT	randomized controlled trial
ALD	alcoholic liver disease
